# Osteoarthritis, bronchial asthma, and periodontitis: is there a mutual influence? A 5-year case–control study

**DOI:** 10.1186/s12903-025-07086-9

**Published:** 2025-12-26

**Authors:** Layal Bou Semaan, Khushboo Kalani, Muhammad H. A. Saleh, Bidisha Ray, Parth Ghataliya, Hend Abulatifa, Hom-Lay Wang

**Affiliations:** 1 Department of Periodontology, University of Alabama, Birmingham School of Dentistry, Birmingham, AL USA; 2 Department of Periodontics and Oral Medicine, The University of Michigan School of Dentistry, Ann Arbor, MI USA

**Keywords:** Periodontitis, Diseases, Risk factor assessment, Health Care Survey, Humans, Asthma, Periodontal diseases, Osteoarthritis

## Abstract

**Objective:**

To investigate the influence of osteoarthritis (OA), bronchial asthma (BA), and the concomitant presence of both conditions on the progression of periodontitis over a five-year follow-up in patients under periodontal maintenance care.

**Methods:**

This case–control longitudinal cohort study analyzed 276 patients divided into four age- and gender-matched groups. Healthy (no OA, no BA), OA, BA, and BAOA (both conditions), with 69 patients in each group. Baseline periodontal parameters, tooth loss due to periodontitis, number of deep pockets (≥ 5 mm), and additional treatment needs were assessed and compared across groups over a mean follow-up duration of 6.5 ± 2.2 years.

**Results:**

At baseline, the Healthy group exhibited significantly more periodontal pockets ≥ 5 mm (11.4 ± 13.1) compared to the OA (4.6 ± 11.0), BA (2.8 ± 5.1), and BAOA (3.9 ± 7.6) groups (*p* < 0.001). However, this difference was not significant after adjusting for confounders (*p* = 0.715). Tooth loss due to periodontitis during follow-up was also not significantly different among groups (*p* = 0.169). The need for invasive treatments was lower in OA and BAOA patients compared to Healthy patients, but this trend did not reach statistical significance (OR = 0.39, *p* = 0.063 for OA; OR = 0.44, *p* = 0.092 for BAOA).

**Conclusion:**

The presence of OA and/or BA does not appear to significantly influence the progression of periodontitis when controlling for confounding variables in a maintained patient population.

**Supplementary Information:**

The online version contains supplementary material available at 10.1186/s12903-025-07086-9.

## Introduction

Periodontitis (PD) is an inflammatory condition caused by dysbiotic microflora, leading to the progressive destruction of the periodontal ligament, cementum, and alveolar bone [1] In 2021, over 1 billion people had severe periodontitis, with a global age-standardized prevalence of 12.50%, and by 2050, cases will rise by 44.32%, exceeding 1.5 billion [1]. Recent studies indicate that systemic diseases such as diabetes [2], cardiovascular diseases [3], asthma (BA) [4], rheumatoid arthritis (RA) [5], osteoarthritis [6], some cancers [7], chronic inflammatory conditions [8], and periodontitis are interconnected, suggesting shared inflammatory pathways and potential bidirectional influences.

Osteoarthritis (OA) is the degeneration of cartilage and bone, often resulting in disability that may require joint replacement [9]. Research has shown that inflammation is a crucial factor in OA progression [6]. OA and periodontitis share similarities, including the involvement of inflammatory cytokines like IL-1β, TNF, and IL-6. This inflammatory response releases enzymes that contribute to the breakdown of cartilage in OA and alveolar bone in periodontitis [10, 11]. Studies indicate that periodontal pathogens may enter the knee joints of OA patients, worsening symptoms. Kim et al. linked periodontitis to knee OA, revealing that increased severity raises risk [6]. Over 15 years, Ma et al. noted a 15% higher osteoarthritis risk in periodontitis patients than controls. Additionally, OA patients have a higher risk of periodontitis, likely due to physical limitations affecting oral hygiene and increasing bacterial colonization [12]. Sao et al. identified 72 overlapping differentially expressed genes (DEGs) in RA, OA, and PD, highlighting 15 central hub genes. Findings suggest *P.gingivalis *may worsen inflammatory conditions, proposing eight genes as potential biomarkers or therapeutic targets for RA, OA, and periodontitis management [13].

Studies have identified that individuals with BA have a higher genetic predisposition to developing arthritis, and conversely, arthritis also appears to increase the risk of BA. These associations are likely mediated through shared inflammatory pathways and immune responses, especially involving allergic mechanisms and cytokines like IL-1β, that underpin both conditions [14, 15].

BA is a chronic inflammatory disease of the airways that leads to wheezing, shortness of breath, and coughing. It involves immune cells, prostaglandins, inflammatory cells (eosinophils, T lymphocytes, macrophages, and neutrophils), cytokines (IL-4, IL-5, IL-13, and TNF-α), and nitric oxide, which physiologically participates in bronchial remodeling [16–18]. The relationship between periodontitis and BA is unclear. Some research shows a positive correlation [4, 19], whereas other studies have found periodontitis to be inversely related to BA [20, 21]. A recent study by Saleh et al. found an inverse association between advanced periodontitis and BA, suggesting that BA may protect against severe periodontitis [22].

As observed, the literature lacks consensus regarding the relationship between periodontitis, OA, and BA in adult populations. This study aims to explore the impact of the two inflammatory diseases together or independently on the progression of periodontitis in patients periodontally treated and maintained over more than five years.

## Methods

### Study design and population

This longitudinal retrospective study analyzed clinical data collected from 2013 to 2024 at the University of Michigan School of Dentistry, Ann Arbor, Michigan, USA. Ethical approval for this study was obtained from the University of Michigan Medical School Institutional Review Board (IRBMED: Study eResearch ID: HUM00228878). The study was conducted in accordance with the Helsinki Declaration, and manuscript preparation adhered to the STROBE guidelines (Appendix S1).

The study population included patients diagnosed with OA and BA. Participants were categorized into four groups: (1) Healthy (no OA, no BA), (2) BA (bronchial asthma), (3) OA (osteoarthritis), and (4) BAOA (both BA and OA). A matching procedure was conducted to create homogeneous groups based on age and sex, ensuring comparability across cohorts. The algorithm was designed to maximize the number of included individuals while adhering to a strict age difference constraint of five years or less within a quartile. A total of 276 patients were selected, with 69 individuals per group.

### Inclusion and exclusion criteria

Inclusion criteria comprised patients aged 30–85 years with a complete medical history recorded at baseline, a comprehensive periodontal chart, and full-mouth radiographs of diagnostic quality taken within 12 months of the baseline periodontal examination. A diagnostic quality radiograph was defined as one that provided clear visualization of the lamina dura and alveolar bone crest without overlapping structures to assess periodontal conditions accurately. Patients with a documented history of BA, OA, or both were included. Patients who underwent periodontal maintenance at the University of Michigan School of Dentistry were included [23].

Exclusion criteria included patients with incomplete medical or dental records. Additionally, those diagnosed with other significant respiratory conditions, including chronic bronchitis, chronic obstructive pulmonary disease (COPD), long-term steroid usage, and rheumatoid arthritis, which might confound the study results, were excluded. Diagnoses of BA and OA were self-reported and physician-confirmed through medical questionnaires provided at admission.

### Data collection

Calibrated researchers extracted clinical data from electronic health records (KK, BR, PG, and HAL), recording various parameters for each patient. Demographic information included age (at admission) and sex. Medical history comprised self-reported and physician-confirmed diagnoses of BA and OA, a history of diabetes mellitus, and smoking status, categorized into never-smokers, former smokers (with a washout period of ≥ 15 years), and current smokers. Occasional smoker was allocated for patients smoking up to 10 cigarettes per day; smokers/moderate smokers for those smoking up to 20 cigarettes per day and heavy smoker if > 20 cigarettes were smoked per day. Baseline periodontal status was assessed through the number of teeth present, periodontal pockets (≥ 5 mm), and staging and grading of periodontitis. Periodontitis diagnosis and severity were determined using the 2017 World Workshop Classification of Periodontal and Peri-Implant Diseases and Conditions. The British Society of Periodontology’s implementation (BSP-i) of the 2017 World Workshop Classification was utilized [24]. At the patient level, periodontitis was classified based on staging (I, II, III, IV) and grading (A, B, C) using a single trained investigator (MHAS) [24, 25], relying primarily on radiographic bone loss (RBL).

Periodontitis progression was measured by the number of teeth lost due to periodontitis (TLP) over the follow-up period [23], the final count of periodontal pockets (≥ 5 mm) at the last visit, frequency of SPT visits per year, and the need for additional periodontal treatment, including scaling and root planning (SRP) or surgical intervention. The follow-up period varied among patients, ranging between approximately 1 and 10 years, depending on individual maintenance records.

### Statistical analysis

Descriptive statistics were performed for categorical variables using absolute and relative frequencies, while continuous variables were summarized using mean, standard deviation (SD), range, median, and interquartile range (IQR). Group comparisons were conducted using Chi-square (χ2) tests for categorical variables and one-way ANOVA (F-tests) for continuous variables to assess homogeneity between groups regarding smoking status, diabetes, follow-up duration, and periodontal maintenance frequency.

One-way ANOVA models were employed to compare disease-related variables at the first visit, such as periodontitis stage, grade, number of teeth, number of periodontal pockets (≥ 5 mm), and these models were adjusted for smoking and diabetes as potential confounders. Ordinal binary regression models were applied for categorical disease-related variables, with adjusted odds ratios and 95% confidence intervals (CIs) obtained from Wald’s Chi2 statistic. A test of parallel lines was performed to validate the proportional odds assumption.

The longitudinal progression of periodontitis was assessed using multifactorial ANOVA models, examining the number of TLP, the final number of deep pockets, and additional treatments required (SRP or surgical interventions). These models were adjusted for potential confounders, including smoking, diabetes, follow-up duration, and SPT frequency. Ordinal logistic regression was used to evaluate factors influencing the need for invasive periodontal treatments.

#### Sample size and power analysis

A post-hoc power analysis was conducted to evaluate the adequacy of the sample size. With 276 patients (approximately 69 per group), the study demonstrated 88.5% power at the 5% significance level to detect a medium effect size (f = 0.25) in a one-way ANOVA across four groups. In this context, the “effect” refers to the difference in mean number of teeth lost due to periodontitis between groups; for example, an effect size of f = 0.25 corresponds to group means of 0.1, 0.3, 0.5, and 0.7, assuming a standard deviation of 1.0. The significance level for all statistical analyses was set at α = 0.05.

## Results

### Patient demographics

This study included 276 patients, with 69 per group (Healthy, BA, OA, and BAOA) (Table 1). The groups were matched by age and sex, with a mean age of 63.1 ± 11.9 years (range: 32–85 years) and a sex distribution of 96 males (34.8%) and 180 females (65.2%).

**Table 1:** Patient Demographics

Characteristic	Normal	BA	OA	OA + RA	Total	
Number per group, n (%)	69 (25%)	69 (25%)	69 (25%)	69 (25%)	276 (100%)	MATCHED
Gender						MATCHED *p* = 1.000
Male, n (%)	24 (34.8)	24 (34.8)	24 (34.8)	24 (34.8)	96 (34.8%)	
Female, n (%)	45 (65.2)	45 (65.2)	45 (65.2)	45 (65.2)	180 (65.2%)	
Age (years)						MATCHED *p* = 0.999
Mean ± SD	62.9 ± 11.2	63.2 ± 11.4	63.0 ± 10.9	63.2 ± 11.1	63.1 ± 11.9	
Smoking status, n (%)	276 (100.0)					*p* = 0.004
No	45 (65.2)	43 (62.3)	22 (31.9)	31 (44.9)	146 (52.9)	
Former	10 (14.5)	17 (24.6)	27 (39.1)	18 (26.1)	72 (26.1)	
Current	9 (13.0)	9 (13.0)	20 (29.0)	20 (29.0)	58 (21.0)	
Smoking + level, n (%)						*P* < 0.001
No	44 (75.9)	43 (62.3)	22 (47.8)	37(55.2)	146 (60.8)	
Former light	6 (10.3)	11 (15.9)	1 (2.2)	9 (13.4)	27 (11.3)	
Former Heavy	1 (1.7)	3 (4.3)	12 (26.1)	6 (9.0)	22 (9.2)	
Current Light	5 (8.6)	10 (14.5)	5 (10.9)	8 (11.9)	28 (11.7)	
Current Heavy	2 (3.4)	2 (2.9)	6 (13)	7 (10.4)	17 (7.1)	
Diabetes, n (%)						*p* = 0.510
No	56 (81.2)	52 (75.4)	59 (85.5)	52 (75.4)	223 (80.8)	
Yes	13 (18.8)	17 (24.6)	10 (14.5)	17 (24.6)	53 (19.2)	
Periodontitis, n (%)	69 (100)	69 (100)	69 (100)	69 (100)	276 (100)	MATCHED
Stage, n (%)						*p* = 0.001
1	8 (12.5)	29 (42.0)	17 (24.6)	22 (33.3)	76 (28.4)	
2	46 (66.7)	26 (37.7)	49 (71.0)	42 (63.6)	172 (64.2)	
3	14 (20.3)	4 (5.8)	2 (2.9)	2 (3.0)	17 (6.3)	
4	1 (1.4)	1 (1.4)	1 (1.4)	0 (0.0)	3 (1.1)	
Grade, n (%)						*p* = 0.810
A	44 (63.8)	21 (30.4)	19 (27.5)	46 (69.7)	175 (65.3)	
B	21 (30.4)	28 (40.6)	18 (26.1)	17 (25.8)	77 (28.7)	
C	4 (5.8)	20 (29.0)	32 (46.4)	3 (4.5)	16 (6.0)	
No. of teeth at first visit						*p* = 0.018
Mean ± SD	26.3 ± 3.2	26.8 ± 3.4	24.9 ± 3.6	24.8 ± 4.0	25.5 ± 3.9	
No. of pockets ≥ 5 mm at first visit						*P* < 0.001
Mean ± SD	11.4 ± 13.1	2.8 ± 5.1	4.6 ± 11.0	3.9 ± 7.6	5.7 ± 10.3	
Follow-up, years Mean ± SD	7.7 ± 2.3	6.3 ± 1.8	5.9 ± 2.1	6.0 ± 2.2	6.5 ± 2.2	*P* < 0.001
SPT visits per year, median (range)	2.0 (0–9)	1.0 (0–7)	0.8 (0–4)	0.3 (0–2)	1.2 (0–9)	*p* < 0.001

The distribution of smoking status varied significantly among the four study groups (*p* = 0.004). The Healthy and BA groups had the highest proportion of non-smokers, while the OA and BAOA groups had the highest proportion of former and current smokers. Specifically, the percentage of non-smokers was 65.2% in the Healthy group, 62.3% in the BA group, 31.9% in the OA group, and 44.9% in the BAOA group. The proportion of former smokers was highest in the OA group (39.1%), followed by BAOA (26.1%), BA (24.6%), and Healthy (14.5%). Current smokers were recorded more in the OA group (29.0%) and were least common in the Healthy group (13.0%). Among current smokers, the proportion of heavy smokers was significantly higher in the OA group (13.0%). In both the OA and BAOA groups, the rate of current heavy smokers exceeded 10%. The Healthy and BA groups had a higher percentage of light smokers (8.6% and 14.5%, respectively), while heavy smokers were more prevalent in the OA group (13.0%) and BAOA group (10.4%).

The prevalence of diabetes was 19.2% in the total sample, with the highest rate in the BA and BAOA groups (24.6%) and the lowest in the OA group (14.5%). However, group differences were not statistically significant (*p* = 0.148).

The mean follow-up duration for the total study population was 6.5 ± 2.2 years (range: 1.1–9.7 years). The mean SPT visits per year for the total population were 1.2. It also varied significantly (*p* < 0.001). Healthy patients had the highest median number of visits (2.0 per year, range: 0–9), while BAOA patients had the lowest frequency of SPT (median: 0.3 visits per year, range: 0–2). The SPT frequency for BA and OA patients is 1.0 and 0.8, respectively.

### Initial periodontal status

**Fig. 1:**
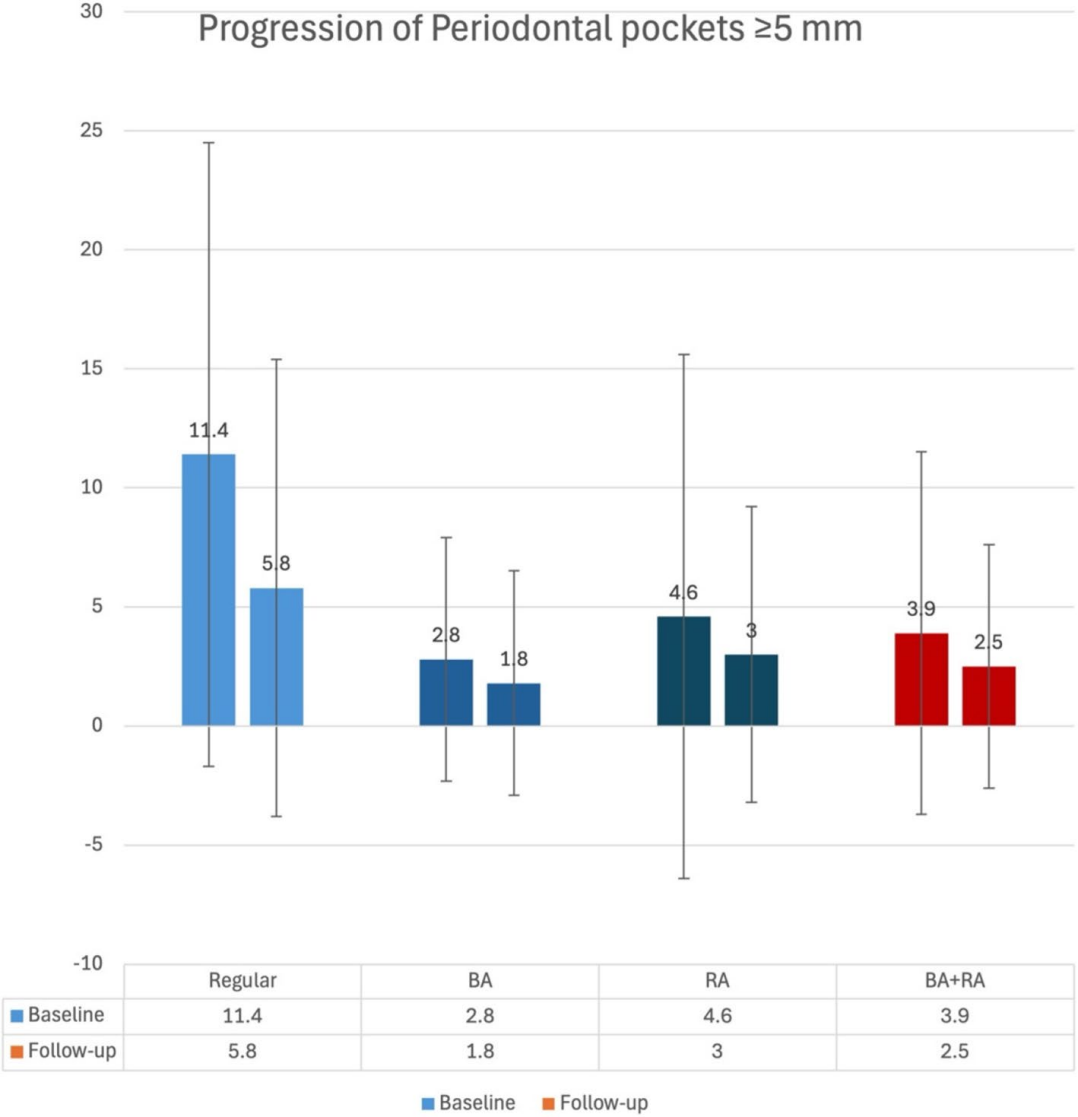
Progression of Periodontal Pockets (≥ 5 mm) by Group. The bar chart illustrates the mean number of periodontal pockets ≥ 5 mm at baseline and follow-up across four groups: Healthy, BA, RA, and BA + RA. At baseline, the Healthy group showed the highest prevalence of deep pockets (11.4), followed by RA (4.6), BA + RA (3.9), and BA (2.8). At follow-up, reductions were observed across all groups, with the Healthy group remaining the highest (5.8), followed by RA (3.0), BA + RA (2.5), and BA (1.8)

The mean number of teeth differed significantly between groups (*p* = 0.018), with OA and BAOA patients presenting with a lesser number of teeth than the Healthy group (Fig. 1). Smoking has a more significant influence on tooth loss compared to all other factors (*p* = 0.024) (Table 2). However, after adjusting for confounders, this difference was no longer significant (*p* = 0.279),

**Table 2:** Number of teeth present at 1 st visit, Number of deep pockets at 1 st visit, Number of lost teeth by periodontitis, and Number of deep pockets at last visit by Group and other independent factors and covariates: Results of multifactorial ANOVA

Number of teeth present at 1 st visit	*p*-value	Number of deep pockets at 1 st visit	*p*-value	Number of lost teeth by Perio	*p*-value	Number of deep pockets at last visit	*p*-value
GROUP	0.279	**GROUP**	< 0.001***	**GROUP**	0.746	**GROUP**	0.715
SMOKING	0.024*	**SMOKING**	0.13	**SMOKING**	0.783	**SMOKING**	0.156
DIABETES	0.379	**DIABETES**	0.038*	**DIABETES**	0.742	**DIABETES**	0.064
FOLLOW UP	0.73	**FOLLOW UP**	0.716	**FOLLOW UP**	0.393	**FOLLOW UP**	0.713
				**STAGE**	0.33	**STAGE**	0.157
				**GRADE**	0.275	**GRADE**	0.389
				**# MAINTENANCES PER YEAR**	0.625	**# MAINTENANCES PER YEAR**	0.889
						**# DEEP POCKETS at 1st**	0.001**

^*^p < 0.05

**p < 0.01

***p < 0.001

The number of periodontal pockets (≥ 5 mm) also differed significantly between groups (*p* < 0.001), with Healthy patients showing the highest number of deep pockets. Multiple pairwise comparison analyses showed significant differences between Healthy and all other groups (*p* < 0.001 for BA and BAOA, *p* = 0.002 for OA) (Table 3). In contrast, no significant differences were observed among the BA, OA, and BAOA groups. Diabetes was significantly associated with the number of pockets (≥ 5 mm) (*p* = 0.038), with diabetic patients presenting an average of 3.3 more pockets than non-diabetic patients (Table 2).

**Table 3:**
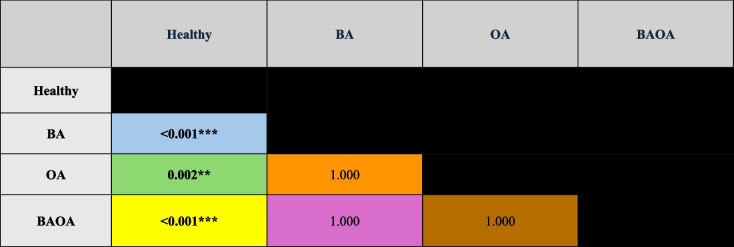
Number of deep pockets at 1^st^ visit by Group and other independent factors and covariates: Results of multiple pairwise comparisons of Bonferroni

Legend (pairwise color-coding) :  Healthy ↔ BA  Healthy ↔ OA  Healthy ↔ BAOA  BA ↔ OA  BA ↔ BAOA  OA ↔ BAOA

^*^p < 0.05

**p < 0.01

***p < 0.001

### Periodontitis progression

#### Tooth Loss Due to Periodontitis (TLP)

The mean total number of TLP during the follow-up period was not significantly different among groups (*p* = 0.169). However, BA patients showed the lowest number of lost teeth. Adjusting for covariates did not alter this outcome (*p* = 0.746) (Table 2).

#### Changes in periodontal pockets (≥ 5 mm)

At the final visit, differences in mean number of pockets (≥ 5 mm) (Fig. 1) remained significant before adjustment (*p* = 0.003) (Table 4). However, after adjusting for baseline periodontal status, the differences observed between the groups were rendered non-significant. (*p* = 0.715). The number of pockets at baseline was the most significant covariate affecting the mean number of pockets ≥ 5 mm at the last follow-up visit (*p* = 0.001). Additionally, the presence of diabetes was a significant contributing factor (*p* = 0.064), with diabetic patients exhibiting an average increase of 2.2 pockets by the last follow-up visit (Table 2).

**Table 4:** Progression and final status of periodontitis by Group: Results are based on one-way ANOVA (F test) for continuous outcomes (# lost teeth, # deep pockets) and Chi-square test (χ2) for categorical outcomes (additional treatment)

OUTCOME	*p*-value
# LOST TEETH BY PERIO	0.169 (F)
# DEEP POCKETS (> = 5 mm)	0.003** (F)
ADDITIONAL TREATMENT (NO TREATMENT/SRP/SURGERY)	< 0.001*** (Chi^**2**^**)**

^*^p < 0.05

**p < 0.01

***p < 0.001

### Need for additional periodontal treatment

The proportion of patients requiring additional periodontal treatment, including SRP or periodontal surgery, differed significantly between groups (*p* < 0.001) (Table 4; Supplemental Figure 1).

Multiple logistic regression suggested that OA and BAOA patients may have lower odds of undergoing invasive periodontal treatments compared to Healthy patients; however, these associations did not reach statistical significance (OR = 0.39, *p* = 0.063 for OA; OR = 0.44, *p* = 0.092 for BAOA). Similarly, patients with Stage II periodontitis showed a trend toward higher odds of requiring surgical treatment compared to those with Stage I, but this was also not statistically significant (OR = 2.05; *p* = 0.072) (Table 5).

**Table 5:** Additional treatment by Group and other independent factors and covariates: Results of multiple ordinal logistic regression (OR and 95%CI, *p*-value)

	OR	95% CI	*p*-value
GROUP			0.102
Healthy	1		
BA	0.68	0.27 - 1.72	0.416
OA	0.39	0.14–1.05	0.063
BA+OA	0.44	0.17–1.14	0.092
SMOKING			0.512
No	1		
Former L	0.84	0.32–2.21	0.723
Former H	0.49	0.15 −1.61	0.241
Current L	1.05	0.37–2.97	0.928
Current H	0.92	0.27–3.10	0.888
DIABETES			
No	1		
Yes	1.22	0.59–2.52	0.595
FOLLOW UP	1.01	0.88–1.15	0.927
STAGE			0.212
1	1		
2	2.05	0.94–4.48	0.072
3–4	1.92	0.31–12.0	0.487
GRADE			
A	1		0.750
B	0.82	0.39–1.75	0.614
C	0.94	0.17–5.17	0.947
# MAINTENANCES PER YEAR	1.01	0.81–1.24	0.959
# DEEP POCKETS at 1st	1.02	0.98–1.06	0.322

*p<0.05

**p<0.01

***p<0.001

## Discussion

This study aimed to assess the effect of OA and BA on the status and progression of periodontitis. The results indicate that while these conditions influence periodontal parameters at baseline, their impact on disease progression is less pronounced after periodontal therapy is provided and these patients are under regular SPT. Despite careful matching by age and sex, some heterogeneity was observed in smoking status and SPT frequency.

OA patients had fewer teeth at baseline, which may be due to smoking, a known confounder in periodontitis progression among arthritic patients [26]. They also had fewer SPT visits, likely due to reduced dexterity and accessibility, as noted by Kim et al. [6]. Even with these risk factors, no significant differences in periodontal progression were found after adjusting for smoking and diabetes, challenging the notion that OA exacerbates periodontitis, unlike rheumatoid arthritis, which shows stronger inflammatory involvement [27].

The study showed BA patients lost fewer teeth due to periodontitis compared to Healthy patients, but this difference wasn't statistically significant. BA patients also had fewer SPT visits, indicating that their disease progression was similar to the control group despite less care. The Mendelian randomization analysis by Jiao et al. [28] suggests no significant genetic causality between BA and periodontitis, reinforcing the opinion that BA does not inherently worsen periodontitis progression. Similarly, Saleh et al. found that while an association between BA and periodontitis exists, it may be confounded by external factors such as medication use and oral hygiene habits rather than a direct pathological link [22]. Overall, the dataset presented in this study implies that BA doesn't, by any means, worsen periodontitis progression.

In our study, we used the number of teeth lost, periodontal pockets ≥ 5 mm, and the need for additional periodontal treatment, following the approach adopted in previous investigations [29–33] as these parameters provide objective, reproducible, and clinically meaningful endpoints in long-term follow-up. Staging and grading, although important for baseline classification, were less applicable in retrospective designs due to their static nature and the difficulty of reconstructing them across decades of records. Probing depth was emphasized because residual deep pockets are consistently the strongest predictors of future breakdown and tooth loss, in line with the treat-to-target concept [33], whereas clinical attachment level is more prone to variability and bleeding on probing, though indicative of inflammation, lacks specificity for long-term progression unless widely distributed.

Patients with OA and BA (BAOA) showed similar results compared to individual conditions. The BAOA group had fewer periodontal pockets (≥ 5 mm) at baseline and less need for surgical intervention than the BA and OA groups. Although the number of periodontal pockets (≥ 5 mm) differed significantly across groups initially, this difference became insignificant after adjusting for baseline pockets, indicating that initial disease severity primarily influenced final outcomes rather than OA or BA presence. This supports findings that baseline periodontal status strongly predicts disease progression [29, 33].

OA and BAOA patients required significantly fewer invasive periodontal treatments than controls, an unexpected finding given the typical link between systemic inflammation and worse periodontal outcomes [32]. This may be due to, as shown in previous studies, the anti-inflammatory effects of medications like corticosteroids and NSAIDs [34, 35]. Monadi et al. found significant bone mineral density loss in asthmatic patients under inhaled corticosteroids [36]. Reduced salivary flow and altered oral microbiota may contribute to compromised periodontal health [37]. Further research is needed to clarify whether these effects are pharmacological or related to better healthcare access.

Smoking was prevalent among both OA and BAOA groups, particularly highlighted by a higher proportion of former heavy smokers in the OA group. As a well-documented risk factor for periodontitis, smoking can increase susceptibility to the disease up to four times via a dose-dependent mechanism [38]. Notably, a recent study revealed that former smokers with asthma are more than twice as likely to develop periodontitis compared to those who have never smoked [22] In contrast, current asthmatics showed lower odds of severe periodontitis compared to never-smokers, which may suggest a potential protective mechanism as discussed earlier [21]. Furthermore, individuals with arthritis and a smoking history exhibited significantly higher odds of developing severe periodontitis than non-smokers, indicating that chronic smoking may cause enduring immunological changes in periodontal tissues [39]^.^

However, the prevalence of diabetes remained similar between groups, despite its well-known role in periodontitis [40]. Diabetic patients presented with a greater number of deep periodontal pockets at the baseline and follow-up, which reinforces the well-established link between poor glycemic control and periodontal destruction [2, 41, 42]. While OA and BA contribute to systemic inflammation, their effect on periodontitis appears less significant than the metabolic dysregulation caused by diabetes. This supports evidence that diabetes is a major determinant of periodontitis progression, surpassing the influence of OA and BA. Notably, the mean SPT frequency across the cohort was only 1.2 visits per year. Previous evidence indicates that while one annual visit can significantly reduce tooth loss [43, 44], optimal periodontal stability, particularly in high-risk patients such as those with diabetes, requires 3–4 visits per year [25, 45]. Thus, more frequent, risk-tailored SPT could mitigate disease progression irrespective of systemic status.

Interestingly, when comparing our OA cohort with epidemiologic data, the prevalence of severe periodontitis (stages III–IV) was lower than in other populations. For example, Rodríguez-Lozano et al. [46] reported a 12.1% prevalence in their OA control group, higher than in our cohort. Differences in study population and design may explain this, as our cohort included patients under supportive periodontal therapy (SPT) with regular maintenance care, potentially stabilizing their periodontal condition. Large-scale epidemiologic data also indicates that among healthy individuals, severe periodontitis prevalence is around 10–12%, with a rising trend [1, 47]. Thus, while our lower rates may reflect specific features of our study population rather than a true deviation from global estimates, this point should be acknowledged as a limitation and considered in the interpretation of our results.

### Limitations and future directions

This study has several limitations. Firstly, the retrospective design hampers our ability to establish causal relationships between BA, OA, and periodontitis progression. Although we found statistical associations, the order of exposures and outcomes is uncertain. Also, BA and OA classification were based on self-reported diagnoses validated by medical records, which might introduce biases related to recall. Secondly, our dataset did not differentiate BA subtypes or evaluate disease severity, oversimplifying the relationship between BA and periodontal outcomes. This could obscure associations between severe or corticosteroid-dependent BA and periodontitis. Thirdly, despite a matched design considering age and sex, there was variability in smoking status and SPT frequency among groups, potentially affecting outcomes. Smoking is a known risk factor for periodontitis. Fourth, reliance on electronic health records (EHRs) raises concerns about data completeness and accuracy since dental students enter them. Attempts were made to include patients with thorough records, but selection bias may have excluded those with incomplete data. Fifth, significant factors like obesity, medication use (especially corticosteroids and NSAIDs), and socioeconomic status were inconsistently recorded, potentially influencing systemic disease and periodontal health. In addition, although groups were matched for age, the overall participant age range was wide (30–85 years). Such variability could have introduced dispersion in clinical outcomes due to age-related differences in immune response, healing capacity, and systemic health status. Unlike smoking and diabetes, age was not incorporated as a stratification or adjustment factor in the analysis, which may have influenced the observed associations. Sixth, periodontal progression was assessed using tooth loss, residual pockets ≥ 5 mm, and need for further treatment; however, staging, grading, CAL, and BOP were not systematically incorporated, which may limit comprehensive evaluation. Lastly, this single-center study from the University of Michigan School of Dentistry may limit generalizability and not capture broader demographic or geographic variability in dental populations practices.

Future research should focus on prospective designs with stratification of BA severity, inclusion of medication history, biomarker assessments, and multicenter collaboration to improve generalizability and deepen our understanding of the systemic-periodontal interface.

## Conclusion

This study found that individually or combined, OA and BA did not significantly influence the progression of periodontitis in a periodontally maintained population over time when adjusting for key factors like smoking, diabetes, and baseline disease severity. Baseline periodontal status and diabetes remained the strongest predictors of disease progression.

## Supplementary Information

Supplementary Material 1.

## Data Availability

The datasets used and/or analyzed during the current study are available from the corresponding author on reasonable request.
