# Supplementary material for: Osteoarthritis, bronchial asthma, and periodontitis: is there a mutual influence? A 5-year case–control study
Source: BMC Oral Health. 2025 Dec 26;26:179. doi: 10.1186/s12903-025-07086-9 (PMC12853903; doi:10.1186/s12903-025-07086-9)
Supplement: Supplementary file 1 — Supplementary Material 1. [file 12903_2025_7086_MOESM1_ESM.docx]

**Supplemental Figure 1 - Additional periodontal treatment by group.**
